# Patient-specific tracer activity in MPI SPECT: A hands-on approach

**DOI:** 10.1007/s12350-015-0286-1

**Published:** 2015-10-09

**Authors:** J. D. van Dijk, P. L. Jager, J. P. Ottervanger, C. H. Slump, S. Knollema, J. A. van Dalen

**Affiliations:** Department of Nuclear Medicine, Isala Hospital, Zwolle, The Netherlands; Department of Cardiology, Isala Hospital, Zwolle, The Netherlands; Department of Medical Physics, Isala Hospital, Zwolle, The Netherlands; MIRA Institute for Biomedical Technology and Technical Medicine, University of Twente, Enschede, The Netherlands

## Introduction

Previously, several studies have reported that a decreasing image quality in heavier patients in myocardial perfusion imaging (MPI) using single-photon emission computed tomography (SPECT) can be compensated by using a body-weight-dependent tracer activity or scan time,^1^-^3^ as illustrated in Figure 1. Although we derived and validated a activity-scan-time formula for a conventional SPECT scanner, this formula cannot simply be used for all SPECT scanners.^1^ Differences in detector sensitivity, technical specifications such a collimator design and geometrical detector configuration, and acquisition and reconstruction settings limit the generalizability of the derived formula. Ideally, a tracer activity-scan-time formula should therefore be derived for each SPECT scanner using the method as described previously.^1^ However, this could be technically challenging and is time consuming. In this technical note, we therefore introduce, as a first-order approach, an alternative simplified method to obtain a body-weight-dependent protocol, which can easily be adopted in every day patient care.

**Figuer 1:**
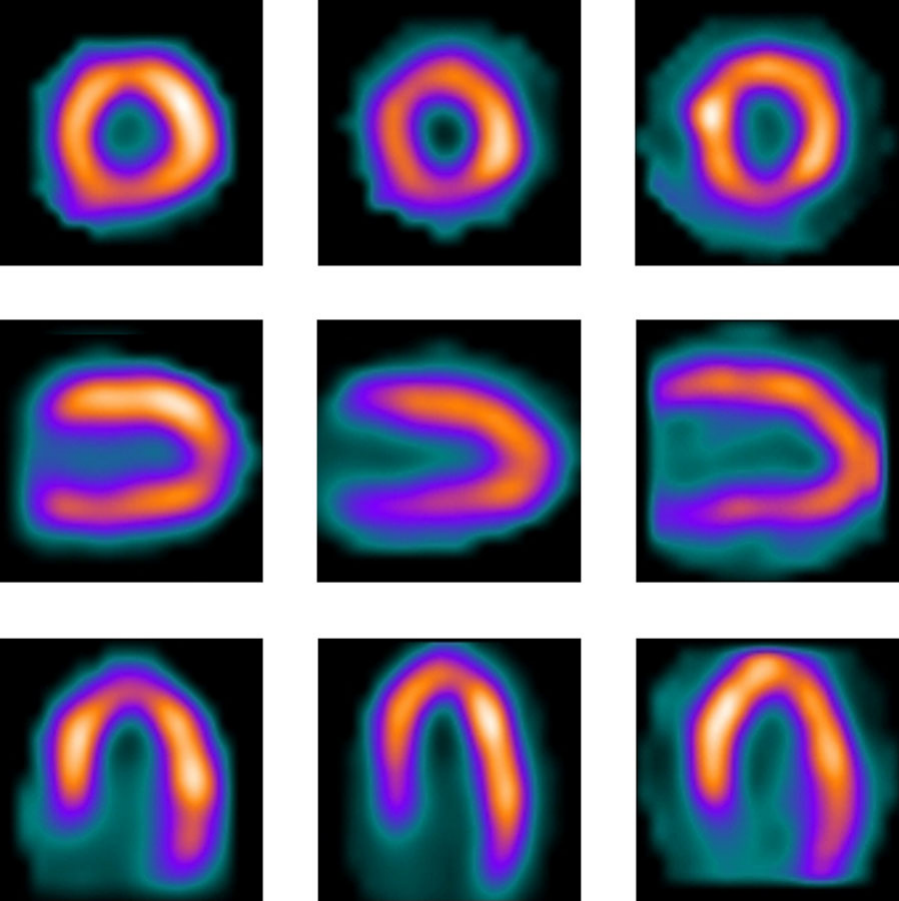
Example of constant image quality in MPI SPECT scans of three male patients without any perfusion defects with varying body weights. From left to right: 66 kg (22.6 kg·m^−2^), 85 kg (25.1 kg·m^−2^), and 124 kg (34.0 kg·m^−2^). The corresponding *short*, *vertical long* and *horizontal long axes* are shown from *top* to *bottom*. A patient-specific tracer activity was applied (330, 395, and 555 MBq, respectively), using a fixed scan time. The image quality of all three sets was scored as ‘good,’ independent of patients’ size

## Deriving a Body-Weight-Dependent Protocol

**Figure 2:**
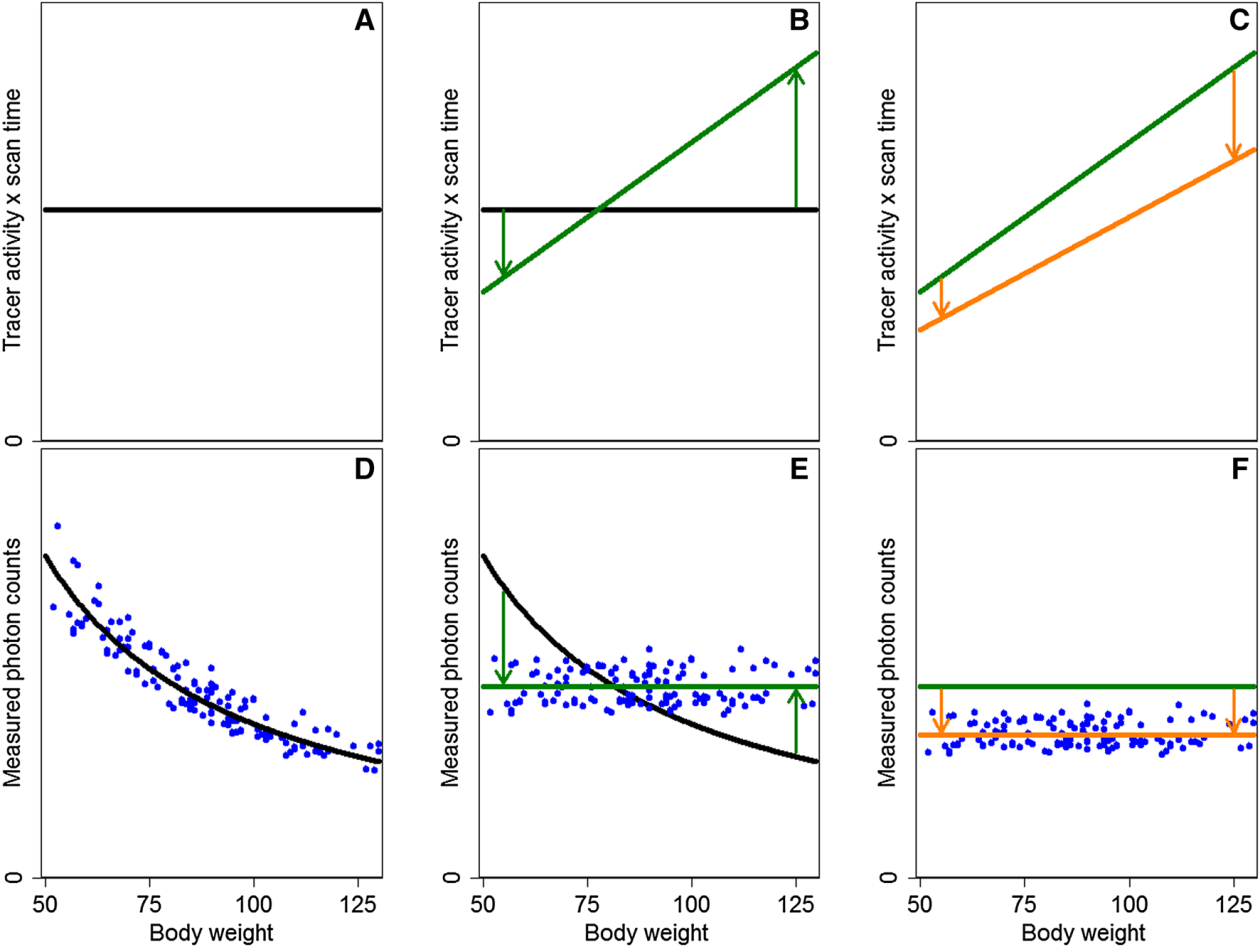
Schematic overview of the transition from a fixed tracer activity and scan-time product (A×T) to a minimized patient-specific A×T. From *left* to *right*: a fixed A×T (**A**) resulting in a decreasing number of photon counts and image quality for heavier patients (**D**). Introduction of a patient-specific A×T (**B**), resulting in a constant number of measured photon counts (**E**). This allows to perform the final step of minimizing the patient-specific A×T (**C**) while maintaining the diagnostic accuracy (**F**). The *dots* represent fictitious data

In cardiac SPECT, the application of a fixed tracer activity and scan-time protocol results in a decreasing number of photon counts in heavier patients due to increased photon attenuation, as demonstrated earlier^1^,^3^ and illustrated in Figure 2A, D. As image quality primarily depends on the number of measured photon counts, a constant number of detected photon counts provides an image quality less dependent on patients’ size.^1^,^3^

A patient-specific protocol will allow obtaining a constant number of detected photons independent of patients’ size.^1^,^3^ A method to derive such a protocol is described recently.^1^ Ideally, the derivation and validation of a patient-specific protocol are performed for each SPECT scanner to account for differences in hardware, software, and acquisition and reconstruction settings. However, to limit the burden of using this extended method, we hereby introduce an alternative, simplified approach, which can easily be adopted in every day patient care. In this approach, we assume that local physicians consider their SPECT image quality of patients with average body weight, AVG_weight_, to be adequate, using the local tracer activity and scan-time combination. To convert this to other patients, a multiplication factor (MF) can be determined using

1a $$ {\text{MF}} = \frac{0.13}{{{{\text{AVG}}_{{\text{weight}}}}^{0.64} }} \times {\text{body}}\;{\text{weight}}\; ( {\text{kg)}} + 1 - 0.13 \times {{\text{AVG}}_{{\text{weight}}}}^{0.36}. $$

This formula is derived from the validated tracer activity and scan-time formula as presented in our recent study by normalizing it to an average patient.^1^ In a patient population with an average body weight of 80 kg, the MF formula can be described by

1b $$ {\text{MF}} = {\text{body}}\;{\text{weight}}\; ( {\text{kg)}} \times 0.0079 + 0.37 $$

In the next step, the body-weight-specific tracer activity or scan time can be calculated using

2a $$ {\text{Patient-specific}}\;{\text{tracer}}\;{\text{activity}}\;({\text{using}}\;{\text{a}}\;{\text{fixed}}\;{\text{scan}}\;{\text{time}}) = {\text{standard}}\;{\text{activity}} \times {\text{MF}} $$

2b $$ {\text{Patient-specific}}\;{\text{scan}}\;{\text{time}}\;({\text{using}}\;{\text{a}}\;{\text{fixed}}\;{\text{tracer}}\;{\text{activity}}) = {\text{standard}}\;{\text{scan}}\;{\text{time}} \times {\text{MF}} $$

As can be seen, MF is 1.0 for a patient of 80 kg when applying Eq. 1b. In that case, the patient-specific tracer activity (or scan time) is the same as the standard administered activity (or scan time). For heavier patients MF is higher than 1, and for less heavy patients it is lower than 1. Table 1 shows an example with the outcome of these equations in practice. The suggested MF is only eligible for conventional SPECT cameras^1^ and patients weighing between 60 and 130 kg, as weights outside this range were not used in deriving the formula.^1^ One could worry that the application of a patient-specific tracer activity or scan-time protocol deviates from the current guidelines.^4^,^5^ However, these guidelines are relatively old and partly outdated due to technological advances and revised insights. Motivated deviation can therefore be justified.

**Table 1:** Multiplication factors to adjust the tracer dose or scan time per projection angle as a function of patient’s weight, using Eq. 1b. Furthermore, two examples for introducing either a patient-specific tracer activity or scan-time protocol are shown, using a scan time of 20 seconds per projection angle (using 32 projections) or a standard tracer activity of 370 MBq, respectively

Body weight	Multiplication factor	Patient-specific activity in MBq using a fixed scan time of 20 seconds (mCi)	Patient-specific scan time (seconds) using a fixed activity of 370 MBq (10 mCi)
60	0.83	307 (8.3)	17
70	0.92	340 (9.2)	18
80	1.00	370 (10.0)	20
90	1.08	400 (10.8)	22
100	1.15	426 (11.5)	23
110	1.23	455 (12.3)	25
120	1.30	481 (13.0)	26
130	1.36	503 (13.6)	27

## Beneficial Effect of Patient-Specific Tracer Activities

Introducing a body-weight-dependent protocol will not only result in image quality that depends less on patients’ size, it also allows for a reduction in the administered activity and, hence, radiation dose to the patient, as shown in a previous study^6^ and illustrated in Figure 2C, F. Nowadays, leaner patients are generally administered a higher activity than clinically necessary. In heavier patients, the currently applied fixed tracer activity is generally low or at best just sufficient. Implementing a patient-specific protocol will therefore result in a better image quality independent of patients’ size. It might even allow an overall tracer activity or scan-time reduction, without compromising diagnostic accuracy.

## Logistics of a Patient-Specific Tracer Activity or Scan Time

**Figure 3:**
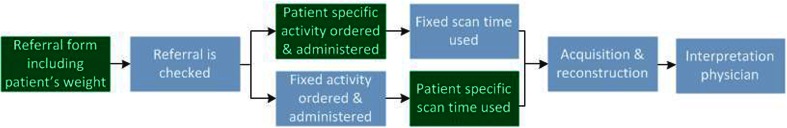
Schematic overview of the required planning and actions to perform when using a patient-specific tracer activity or scan-time protocol. The additional actions that are required as compared to the fixed activity era are indicated in *green*. The MPI-SPECT referral form including patient’s body weight should be checked by a nuclear medicine physician or asked by telephone when booking appointments. Next, either a patient-specific activity should be ordered or a patient-specific scan time should be applied. Subsequently, physicians interpret the reconstructed study and determine whether additional rest imaging is necessary

A schematic overview of the required planning and actions when applying a patient-specific tracer activity or scan-time protocol is shown in Figure 3. Two additional actions are required as compared to the fixed tracer activity era. First, patients’ body weight is always required for planning and should be stated on the requisition or asked by telephone when booking appointments. Second, the activity or scan time must be calculated or derived from an activity-scan-time table and applied to the preparation process of the MPI study.

## Considerations

Prior to introducing patient-specific protocols in clinical practice the following must be considered. First, when using a 1-day stress-first protocol, the administered activity for rest imaging should be more than 2-3 times the stress activity with a delay of 0.5-4 hours between both tracer activity administrations, to allow for sufficient decay of myocardial activity.^4^,^5^ A 2-day protocol prevents this problem and allows the use of identical patient-specific stress and rest activities (when using identical scan times). This will lower the rest activity and, hence, radiation dose, for these patients by a factor 2-3. The use of 2-day protocols can be considered in heavy patients, to reduce the overall radiation dose for both patients and staff. Second, a higher correction factor might be beneficial in patients weighing over 130 kg. However, due to the low number of patients weighting over 130 kg, we were unable to reliably extrapolate the given protocol for these patients. Third, it may be logistically difficult to obtain a variable patient-specific tracer activity for a rest study after the interpretation of the stress scans using a 1-day stress-first protocol. If obtaining variable tracer activities on short notice is difficult, patient-specific scan times (and fixed tracer activity) can be applied alternatively. This, however, may slightly interfere with camera time planning. Fourth, as mentioned above, the shown formulas are a simplified approach and only eligible for conventional SPECT cameras. A different relation between measured photon counts and weight was observed using the newest generation cadmium zinc telluride based SPECT cameras.^3^ Hence, a different activity or scan-time correction should be applied for these scanners.
